# The Coiled Coil and C2 Domains Modulate BCR Localization and BCR-ABL1 Compartmentalization, Transforming Activity and TKI Responsiveness

**DOI:** 10.3390/ijms26146591

**Published:** 2025-07-09

**Authors:** Michele Massimino, Stefania Stella, Chiara Romano, Pietro Buffa, Elena Tirrò, Melissa Drago, Livia Manzella, Cristina Tomarchio, Silvia Rita Vitale, Francesco Di Raimondo, Paolo Vigneri

**Affiliations:** 1 Department of General Surgery and Medical-Surgical Specialties, University of Catania, 95123 Catania, Italy; diraimon@unict.it; 2 Center of Experimental Oncology and Hematology, A.O.U. Policlinico “G. Rodolico-S. Marco”, 95123 Catania, Italy; stefania.stel@gmail.com (S.S.); chiararomano83@gmail.com (C.R.); ele_tir@yahoo.it (E.T.); dragomelissa23@gmail.com (M.D.); manzella@unict.it (L.M.); cristina.tomarchio@hotmail.it (C.T.); silviarita.vitale@gmail.com (S.R.V.); vigneripaolo@gmail.com (P.V.); 3 Department of Clinical and Experimental Medicine, University of Catania, 95100 Catania, Italy; 4 Department of Drug and Health Sciences, University of Catania, 95125 Catania, Italy; pietrobuffa@hotmail.com; 5 Department of Precision Medicine in Medical, Surgical and Critical Care (Me.Pre.C.C.), University of Palermo, 95127 Palermo, Italy; 6 Division of Hematology, A.O.U. Policlinico “G. Rodolico-S. Marco”, 95123 Catania, Italy; 7 Division of Oncology, Humanitas Istituto Clinico Catanese, 95045 Misterbianco, Italy

**Keywords:** BCR-ABL1, TKI, CML, NLS, intracellular localization, BCR, CD34

## Abstract

The BCR-ABL1 chimeric oncoprotein plays a pivotal role in the pathogenesis of Chronic Myeloid Leukemia (CML) as its constitutive kinase activity transforms the hematopoietic stem cell, promoting pro-survival signaling. We and others have previously shown that the manipulation of BCR-ABL1 catalytic activity modulates its intracellular localization, thereby transforming the culprit of CML into a pro-apoptotic protein that selectively kills leukemic cells. Here, we investigated the role of the BCR coiled-coil and C2 domains on BCR-ABL1 intracellular localization and leukemogenic potential. We performed a bioinformatic analysis that identified two putative nuclear localization signals (NLSs) in BCR. Using recombinant DNA strategies, we generated multiple BCR and BCR-ABL1 mutants that were ectopically expressed in human cells. The intracellular localization of each construct was analyzed by immunofluorescence, while their biological activity was investigated employing proliferation and transforming assays. We show that BCR displays two nuclear localization signals functionally inactivated by the coiled-coil and C2 domains. The removal of these regions reactivated the nuclear migration of both BCR and BCR-ABL1 mutants. Moreover, BCR-ABL1 constructs devoid of the coiled-coil and C2 domains displayed reduced transforming potential in Ba/F3 cells and in primary human CD34+ progenitors. Finally, we demonstrate that the deletion of the C2 domain compromises TKI efficacy. Our findings identify two nuclear localization signals in the BCR sequence that are functionally suppressed by the coiled-coil and C2 domains. Targeting these regions may provide additional therapeutic strategies to manipulate both BCR-ABL1 intracellular localization and kinase activity.

## 1. Introduction

The BCR-ABL1 oncogene is the hallmark of Chronic Myeloid Leukemia (CML) as the ensuing chimeric oncoprotein induces the clonal expansion of leukemic cells by activating different downstream targets involved in cell proliferation and survival [1,2].

Despite the unprecedent rates of hematological, cytogenetic and molecular responses achieved with the introduction of multiple ABL1-directed tyrosine kinase inhibitors (TKIs), 20–30% of CML patients fail to achieve long-term benefits from these drugs due to the emergence of resistance mechanisms or because of patient compliance issues [3,4,5]. Moreover, the available evidence suggests that—in a large proportion of patients—even sustained TKI-treatment fails to eradicate the Ph-positive leukemic stem cell [6,7,8,9].

To investigate additional mechanistic strategies that may be employed to eliminate BCR-ABL1 clones, we previously demonstrated that the nuclear entrapment of the leukemic oncoprotein selectively kills Ph-positive cells [10]. Indeed, these findings have led us and different authors to devise pharmacological approaches that induce BCR-ABL1 nuclear accumulation, thereby killing leukemic cells [11,12,13].

ABL1 contributes three Nuclear Localization Signals (NLSs) and a single Nuclear Export Signal (NES) to BCR-ABL1, suggesting that the resulting chimeric oncoprotein should be subjected to bidirectional cytoplasmic–nuclear shuttling [14]. However, multiple groups have shown that BCR-ABL1 presents an exclusively cytoplasmic localization [13]. To date, the contribution of the Breakpoint Cluster Region (BCR) protein to these biological events has remained unclear.

BCR is a multidomain protein characterized by several functionally distinct domains. The N-terminal portion of the gene encodes for a coiled-coil region (CC) and a serine threonine kinase domain (S/TK). The CC is critical for BCR-ABL1 oligomerization, leading to constitutive ABL1 kinase activity, while the S/TK domain contains two SH2 binding sites and a Grb2-interacting site comprising a tyrosine residue in position 177 that is required for BCR-ABL1 transforming activity [15,16,17]. The central portion of the BCR protein is characterized by a Guanine Nucleotide Exchange Factor (GEF) site which binds xeroderma pigmentosum B (XPB) and is involved in DNA repair [18]. The GEF is flanked by a Pleckstrin Homology Domain (PH) and a C2-domain (DC2) implicated in phosphoinositide interaction. Finally, the C-terminal region of BCR displays a RhoGAP (Rho) domain with GTPase activity [19]. Although BCR is reportedly a cytoplasmic protein [20], published evidence has also suggested that BCR associates with different chromosomes during mitosis [21], and that the shorter isoform, BCR p130, can localize to the nucleus [22].

We report here the contribution of the CC and C2 BCR domains to the intracellular localization, transforming the activity and TKI sensitivity of BCR-ABL1.

## 2. Results

### 2.1. Cytoplasmic Localization of BCR Is Dependent on Functionally Inactivated Nuclear Localization Sequences

**Figure 1:**
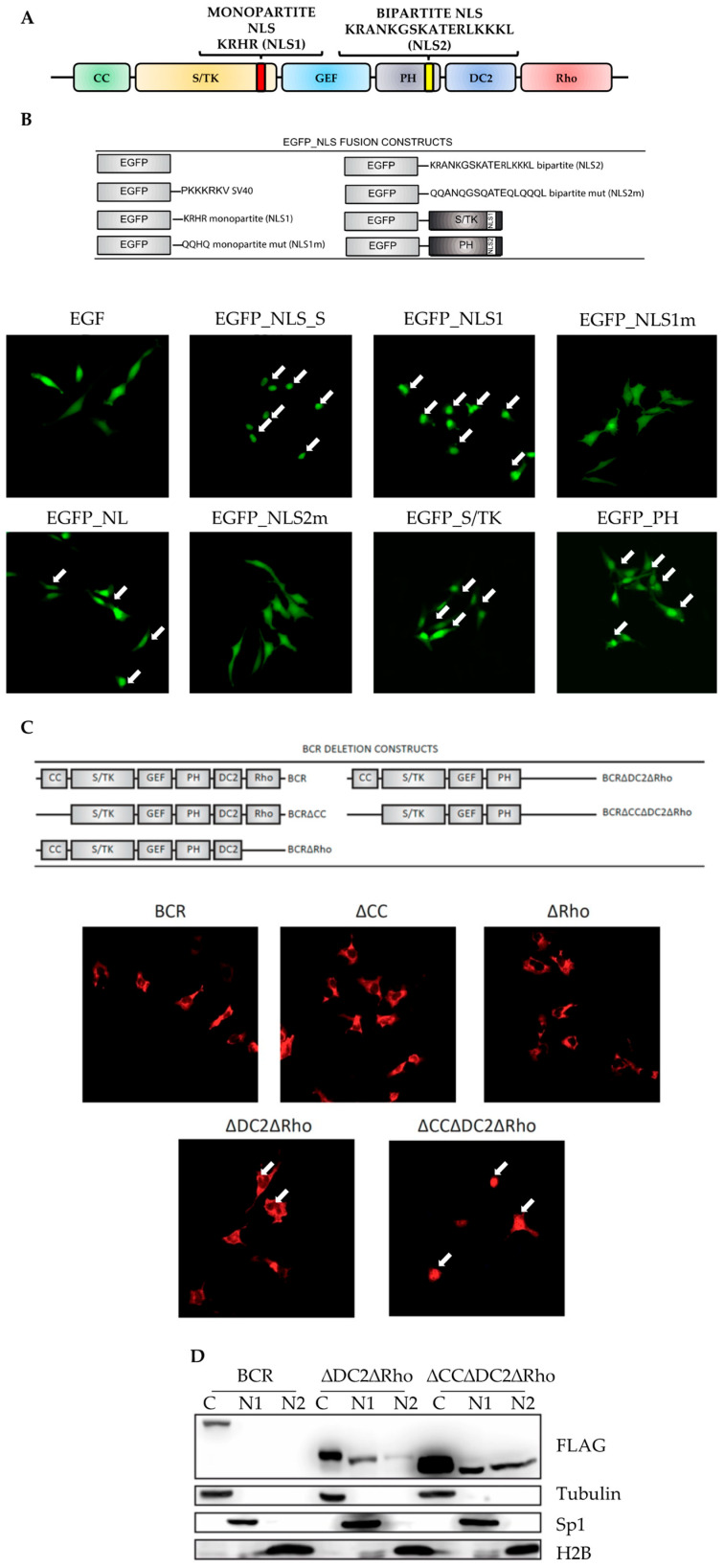
Identification and characterization of two putative nuclear localization signals (NLSs) in the BCR sequence: (**A**) graphic representation of BCR domains and NLSs. NLS1 and NLS2 are monopartite and bipartite, respectively. CC: coiled-coil, S/TK: Serine/Threonine Kinase, GEF: Guanine nucleotide Exchange Factor, PH: Pleckstrin Homology domain, DC2: C2 domain, Rho: RhoGAP domain. (**B**,**C**) (Top panels) images representing the EGFP-NLS (**B**) and BCR (**C**) fusion constructs. NLS1m and NLS2m indicate mutant NLSs, where K and R residues have been substituted with a Q. S/TK and PH indicate the Serine/Threonine kinase and pleckstrin homology domains, respectively. Immunofluorescence of the indicated EGFP ((**B**), bottom panels) fusion constructs and BCR deletion mutants ((**C**), bottom panels) transfected in HeLa cells. (**D**). Cytoplasmic and nuclear fractionation of reported BCR constructs. Tubulin (Tub), histone (H2B) and transcription factor 1 (Sp1) were used to confirm the purity of the nuclear and cytoplasmic fractions. C: cytoplasmic fraction, N1: nuclear fraction, N2: insoluble nuclear fraction.

To investigate the presence of putative nuclear localization signals (NLSs) in the BCR protein, we analyzed its coding sequence using the PredictNLS and P-Sort bioinformatic tools. This analysis identified two alleged NLS: one in the S/TK region and the other in the PH domain (Figure 1A). The first NLS is a single monopartite sequence spanning amino acids 390 to 394 (KRHR). The NLS located in the PH domain displays two sequences (amino acids 802 to 819) separated by a short spacer and should therefore be considered a bipartite NLS (KRANKGSKATERLKKKL).

To analyze if both NLSs could mediate nuclear import, we cloned each individual sequence or the entire S/TK and PH domains in the C-terminal region of an EGFP plasmid, thereby generating the EGFP-NLS1 (monopartite NLS), EGFP-NLS2 (bipartite NLS2), EGFP-S/TK and EGFP-PH constructs. Moreover, to confirm that the EGFP nuclear import was dependent on specific NLS amino acidic residues, we mutagenized all lysines (Ks) and arginines (Rs) in glutamines (Qs) (EGFP-NLS1m and EGFP-NLS2m) [23]. An EGFP plasmid carrying the SV40 NLS was used as a positive control (Figure 1B). Immunofluorescence experiments performed on transfected HeLa cells showed that both NLS1 and NLS2 induced the EGFP nuclear import with an NLS1 more potent than NLS2. The EGFP nuclear import was reduced after the substitution of the R or K residues with Q (EGFP-NLS1m, EGFP-NLS1m), indicating that EGFP nuclear accumulation was dependent on NLS-specific sequences. When we repeated these experiments with the EGFP-S/TK or EGFP-PH plasmids, we observed a consistent nuclear staining in HeLa cells expressing both constructs, supporting the notion that both NLS1 and NLS2 mediate nuclear import.

We then wanted to investigate the intracellular localization of the entire BCR protein and therefore engineered a series of BCR deletion mutants (Figure 1C, upper panel). The overexpression of wild-type BCR-FLAG in HeLa cells resulted in a strong cytoplasmic localization (Figure 1C, lower panel, BCR). Given the results obtained with the EGFP-S/TK and EGFP-PH plasmids, we hypothesized that the BCR’s inability to migrate inside the nucleus may be mediated by the structural conformation of the protein that may result in NLS masking. This phenomenon would inhibit the binding of importin 1, a karyopherin which mediates cytoplasm–nuclear migration [24].

To test this hypothesis, we deleted the coiled-coil domain (BCR^∆CC^), assuming that the oligomerization process may be responsible for NLS inactivation. However, this modification did not result in BCR nuclear localization (Figure 1C, lower panel, ∆CC). We observed the same results when we removed the Rho domain, a region involved in actin polymerization (Figure 1C, lower panel, ∆Rho) [25,26,27]. As the bipartite NLS (NLS2) is localized in the C-terminal portion of the PH domain, we speculated that the C2 domain (DC2) may cause its structural masking. Hence, we removed the DC2 region from BCR^∆Rho^, generating a BCR^∆DC2∆Rho^ construct that displayed weak nuclear staining indicative of NLS2 partial unmasking (Figure 1C, lower panel, ∆DC2∆Rho). To determine if the oligomerization process would affect this phenomenon, we generated a monomeric isoform of BCR^∆DC2∆Rho^, removing the coiled-coil domain (BCR^∆CC∆DC2∆Rho^). Indeed, this construct showed strong nuclear staining (Figure 1C, lower panel, ∆CC∆DC2∆Rho), as confirmed by cytoplasmic–nuclear fractionation experiments (Figure 1D). All together, these findings suggest that the NLS1 and NLS2 in the BCR sequence would be capable of inducing nuclear import, if not for their flanking domains and the coiled coil region that suppress their activity.

### 2.2. The CC and DC2 Contribute to BCR-ABL1 Cytoplasmic Retention

**Figure 2:**
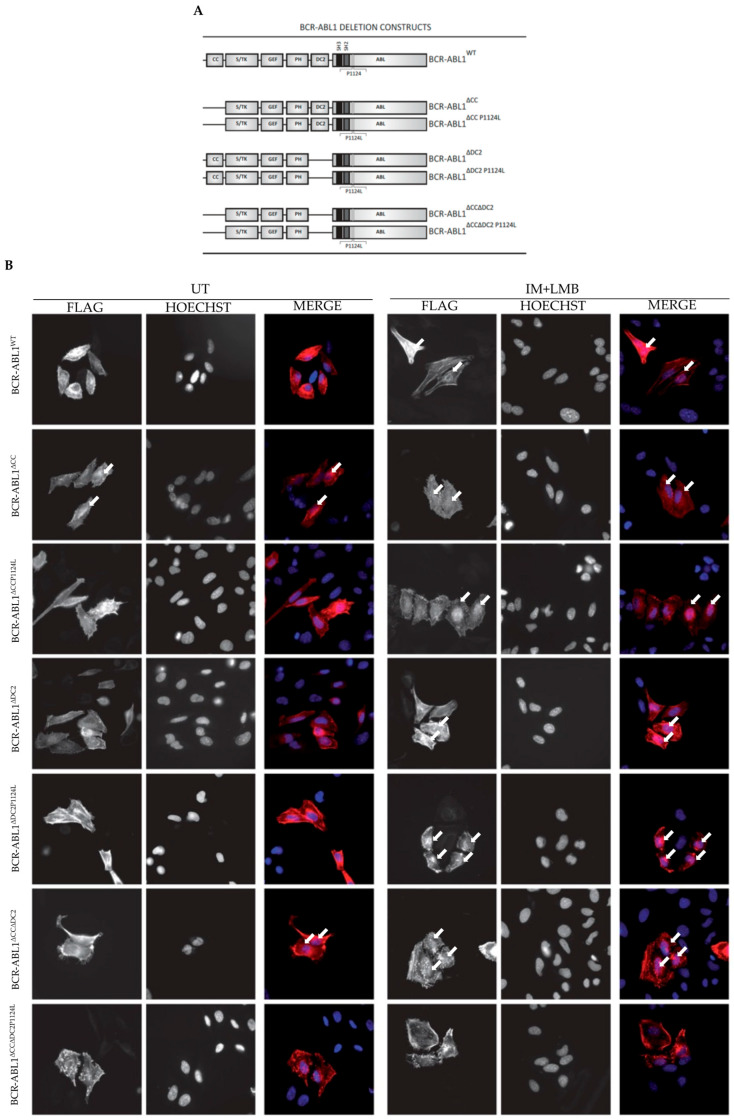
The coiled-coil region and the C2 domain modulate subcellular localization of BCR-ABL1. (**A**) Images representing multiple BCR-ABL1 constructs employed for the depicted experiments. P1124L indicates a proline–leucine substitution in position 1124 of BCR-ABL1. (**B**) Immunofluorescence of transfected HeLa cells with the specified FLAG-tagged BCR-ABL1 mutants. FLAG and Hoechst represent BCR-ABL1 and nuclear staining, respectively. IM+LMB denotes combined treatment with IM (10 µM) and LMB (10 nM) for 24 h. White arrows indicate BCR-ABL1 nuclear migration.

We have previously shown that the BCR-ABL1 oncoprotein is retained in the cytoplasm. We have also demonstrated that the IM-dependent suppression of BCR-ABL1 kinase activity, followed by the pharmacological inhibition of exportin 1 by LMB, induces BCR-ABL1 nuclear accumulation [10]. Since the NLSs in BCR are functionally inhibited by the CC and DC2, we generated BCR-ABL1 constructs lacking these domains and evaluated their intracellular localization after transient transfection in HeLa cells in the presence or the absence of IM and LMB (Figure 2). As expected, the IM+LMB combination induced BCR-ABL1 nuclear accumulation, while the CC deletion that generates a kinase defective BCR-ABL1^∆CC^ caused a weak nuclear staining that was increased after IM and LMB treatment. To further demonstrate that BCR-ABL1^∆CC^ nuclear import was dependent on defective kinase activity, we generated the BCR-ABL1^∆CCP1124L^ mutant in which the proline in position 1124 is substituted by a leucine residue. As previously reported, this monomeric BCR-ABL1 construct retains constitutive tyrosine kinase activity [28]. Indeed, in experiments employing BCR-ABL1^∆CCP1124L^, we observed a reduced nuclear staining that was re-established by IM+LMB, confirming our previously published results that BCR-ABL1 cytoplasmic retention is dependent on its kinase activity. To explore the C2 domain contribution to BCR-ABL1 subcellular localization, we generated BCR-ABL1^∆DC2^ and BCR-ABL1^∆DC2P1124L^. Both mutants showed a weak nuclear localization after IM+LMB treatment, implying that the DC2 deletion modifies the oncoprotein’s structure, strongly reducing IM-mediated reactivation of the NLSs. To investigate the role of the oligomerization process in DC2-dependent BCR-ABL1 cytoplasmic retention, we deleted the CC region from both BCR-ABL1^∆DC2^ and BCR-ABL1^∆DC2P1124L^, obtaining BCR-ABL1^∆CC∆DC2^ and BCR-ABL1^∆CC∆DC2P1124L^, respectively. Interestingly, in BCR-ABL1^∆CC∆DC2^, the CC deletion partially restored BCR-ABL1 nuclear import. However, the introduction of the P1124L substitution re-localized BCR-ABL1^∆CC∆DC2P1124L^ to the cytoplasm even after IM+LMB, supporting the notion that the C2 domain contributes to the kinase-dependent cytoplasmic localization of BCR-ABL1. Given that, drugs potentially able to alter the subcellular localization of BCR-ABL1, thereby impacting the therapeutic response in CML patients [29].

**Figure 3:**
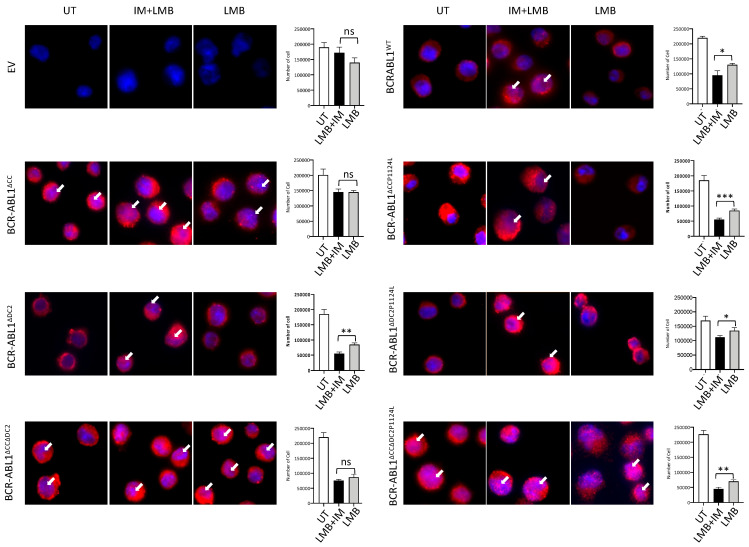
The coiled-coil region and C2 domain contribute to the cytoplasmic entrapment of BCR-ABL1. Immunofluorescence experiments in lentivirally transduced Ba/F3 cells expressing the indicated BCR-ABL1 constructs. FLAG and Hoechst (blue) indicate BCR-ABL1 and nuclear staining, respectively. Empty vector (EV) was used as a control to evaluate non-specific antibody adsorption. Graphs (right panels) indicate the number of living cells in each experimental condition: untreated (white column), exposed to both IM (10 µM) and LMB (10 nM) (IM+LMB) (black column) or to LMB alone (gray column). Bars indicate the standard deviation obtained from two independent experiments conducted in triplicates. *p* values were calculated for IM+LMB vs. LMB alone applying the *t*-test (* *p* < 0.05, ** *p* < 0.01, *** *p* < 0.001). For all pictures, the single channels for each staining are reported in Supplementary Figure S2).

We subsequently explored the role of the CC and DC2 domains on BCR-ABL1 subcellular localization and cell viability using hematopoietic Ba/F3 cells (Figure 3). As expected, Ba/F3s expressing BCR-ABL1^WT^ showed nuclear staining and reduced cell viability after IM+LMB. When we employed different deletion constructs, we found that BCR-ABL1^∆CCP1124L^, BCR-ABL1^∆DC2^, and BCR-ABL1^∆DC2P1124L^ were mostly in the cytoplasm and that exposure to IM and LMB partially re-localized them to the nucleus. Moreover, in these cells, the IM+LMB combination reduced the number of living cells compared to LMB alone (fold reduction: BCR-ABL1^∆CCP1124L^ 1.7, BCR-ABL1^∆DC2^ 1.6, BCR-ABL1^∆DC2P1124L^ 1.2). These data confirm that the nuclear entrapment of a catalytically functional BCR-ABL1 causes cell death and suggest that the deletion of the C2 domain does not influence this effect. Interestingly, we observed a partial nuclear staining of all CC deletion mutants devoid of the DC2: BCR-ABL1^∆CC∆DC2^ and kinase active BCR-ABL1^∆CC∆DC2P1124L^. However, the nuclear accumulation of BCR-ABL1^∆CC∆DC2P1124L^ in untreated cells did not result in cell death, which was only observed after IM+LMB treatment. Finally, BCR-ABL1^∆CC^ and BCR-ABL1^∆CC∆DC2^ did not reduce cell viability after IM+LMB. The cytoplasmic and nuclear distribution of these mutants is reported in Supplemental Table S1.

To exclude that the deletion of the CC and DC2 domains caused the reactivation of the NLSs’ sequence of ABL1, we generated a BCR-ABL1^∆CC∆DC2^ deletion construct where the K and R residues of all three NLS^ABL1^ sequences were mutated in Q (BCR-ABL1^∆CC∆DC2ABLNLSm^). Immunofluorescence experiments with this construct demonstrated that nuclear import was preserved, suggesting that the deletion of the CC and DC2 domains did not restore the activity of the NLSs’ sequence of the ABL1 protein (Supplementary Figure S1).

### 2.3. The DC2 Modulates BCR-ABL1 Tyrosine Kinase Activity and Transforming Potential

**Figure 4:**
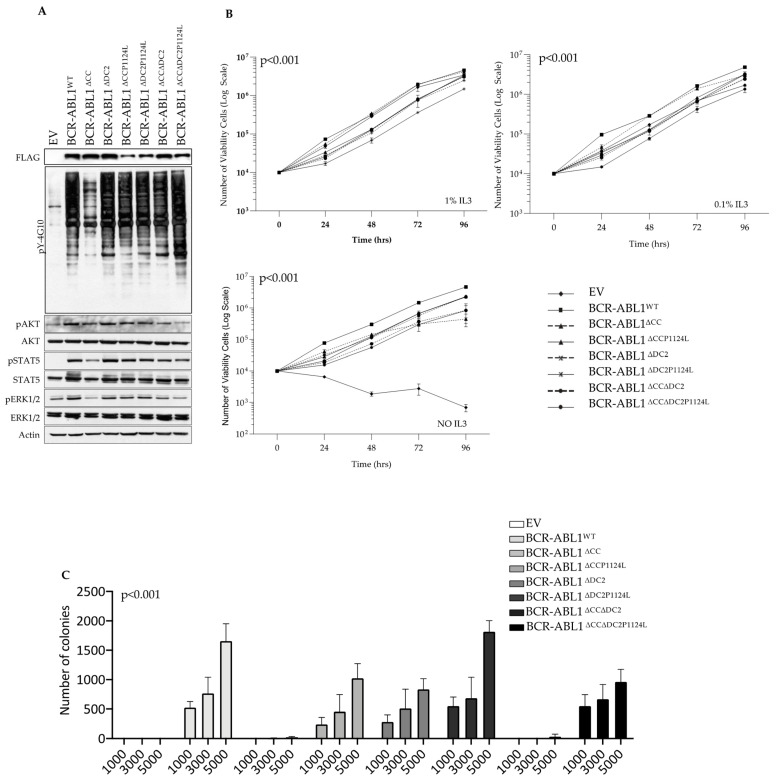
The coiled-coil region and the C2 domain modulate BCR-ABL1 catalytic activity and transforming potential. (**A**) Immunoblots comparing the overall tyrosine kinase phosphorylation levels (pY-4G10) (upper panel) or the specific phosphorylation of Akt, STAT5 and ERK1/2 by the indicated BCR-ABL1 constructs (FLAG). Actin was used as a loading control (bottom panel). (**B**) Proliferation curves of Ba/F3 cells expressing the specified BCR-ABL1 mutants cultivated in the absence or the presence of the indicated IL3 concentrations. (**C**) Histograms depict colony-forming units expressed as number of colonies after 10 days of culture. Numbers below the graph indicate the number of plated cells for each condition. For (**B**,**C**), bars indicate the standard deviation derived from three independent experiments performed in triplicates. *p* values for statistical significance were calculated using variance analysis (Anova) (* *p* < 0.05, ** *p* < 0.01, *** *p* < 0.001).

To establish the role of the C2 domain on BCR-ABL1 transforming potential, we transduced Ba/F3 cells with multiple BCR-ABL1 constructs and measured their ability to phosphorylate known BCR-ABL1 downstream targets (Figure 4). Compared to BCR-ABL1^WT^, the monomeric BCR-ABL1^∆CC^ isoform showed reduced tyrosine phosphorylation, unlike the remaining deletion mutants. Interestingly, BCR-ABL1^∆CC∆DC2^ remained kinase-proficient despite the absence of the CC, indicating that the DC2 deletion restored the catalytic activity of a BCR-ABL1 mutant devoid of the CC region. This effect was further increased when we introduced the P1124L mutation (BCR-ABL1^∆CC∆DC2P1124L^) (Figure 4A, top panel). When we investigated the phosphorylation of well-known BCR-ABL1 downstream targets, we found no detectable differences in phosphorylation levels comparing BCR-ABL1^WT^ to all other deletion mutants excluding the BCR-ABL1^∆CC^ isoform (Figure 4A, bottom panel). We next evaluated the transforming activity of each BCR-ABL1 construct in Ba/F3 cells and found that, in the presence of low IL3 levels, BCR-ABL1^∆CC^ was the only isoform incapable of transforming this cell line. However, upon complete IL3 deprivation (NO IL3), BCR-ABL1^∆CC∆DC2^ was less effective in promoting IL3-independent growth with its full transforming potential restored by the insertion of the P1124L mutation. As expected, BCR-ABL1^∆CC^ failed to induce Ba/F3 cell growth (Figure 4B). Statistically significant differences for each condition are reported in Supplementary Tables S2–S4.

Lastly, we investigated the impact of the BCR-ABL1 deletion mutants on the clonogenic potential of Ba/F3 cells. We found that BCR-ABL1 lacking the DC2 (BCR-ABL1^∆DC2^) showed lower clonogenic potential compared to the wild-type oncoprotein. Moreover, unlike what we observed for the Ba/F3 proliferation rates, the co-deletion of the CC and DC2 regions reduced the overall colony-forming ability, even in the construct displaying the P1124L substitution (Figure 4C).

### 2.4. The DC2 Reduces TKI-Dependent Inhibition of BCR-ABL1 Catalytic and Clonogenic Activity

**Figure 5:**
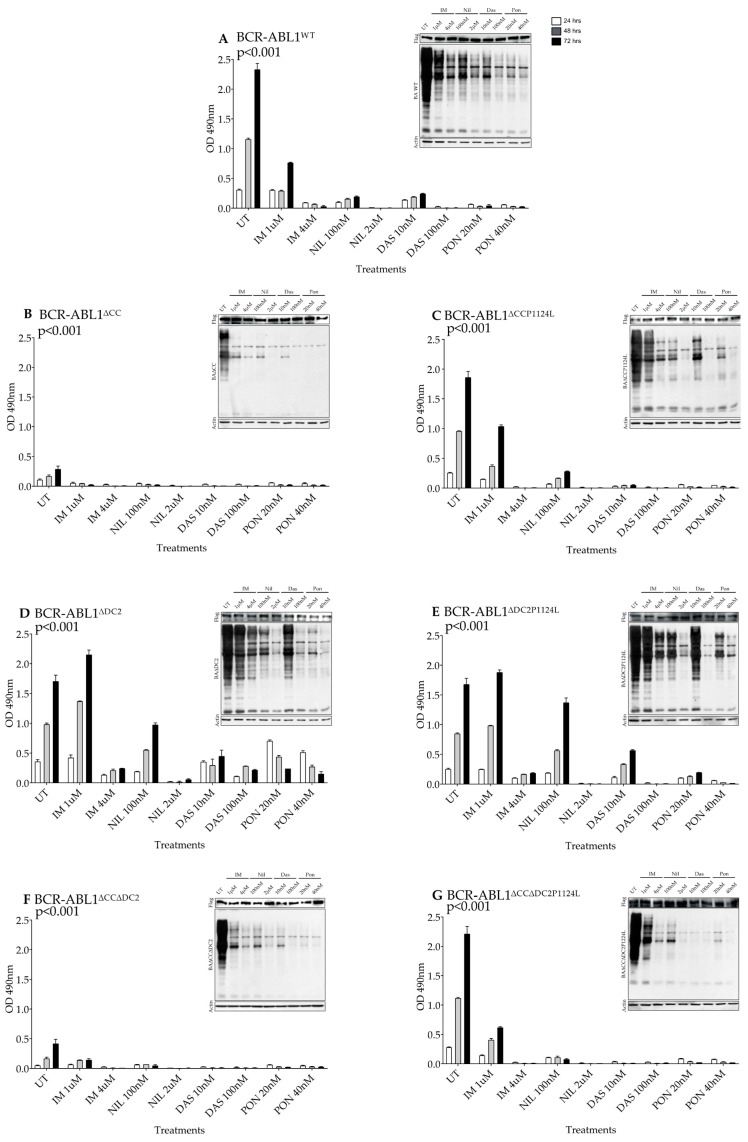
The C2 domain affects BCR-ABL1 TKI response. (**A**–**G**) Ba/F3 cells expressing the specified BCR-ABL1 mutants were either left untreated or exposed to the indicated TKIs at different concentrations. Histograms show cell viability depicted as OD450 nm after 24 (white column), 48 (gray column) and 72 (black column) hours. Bars report standard deviation derived from three independent experiments performed in triplicates. *p* values indicate statistical significance by variance analysis (Anova) (* *p* < 0.05, ** *p* < 0.01, *** *p* < 0.001). Immunoblots (top right insert) indicate the overall BCR-ABL1 construct expression (FLAG) and their respective tyrosine phosphorylation levels in cells left untreated or exposed for 24 h to the specified TKIs.

To investigate the contribution of the C2 domain on BCR-ABL1 TKI responsiveness, we exposed Ba/F3 cells transduced with multiple BCR-ABL1 mutants [30,31,32] to different TKIs and measured their proliferation rates and total intracellular tyrosine phosphorylation (Figure 5). Compared to BCR-ABL1^WT^, BCR-ABL1^∆CC^ was mostly devoid of tyrosine kinase activity (Figure 5A), and the introduction of the P1124L mutation mildly reduced the efficacy of IM (1 µM) and DAS (10 nM) (Figure 5B,C). On the contrary, BCR-ABL1^∆DC2^ maintained its tyrosine phosphorylation that was further increased in BCR-ABL1^∆DC2P1124L^. Both constructs were resistant to IM (1 µM) and displayed a modest reduction of their catalytic activity after NIL (100 nM), with BCR-ABL1^∆DC2^ also displaying reduced sensitivity to DAS and PON (Figure 5D,E). Finally, BCR-ABL1^∆CC∆DC2^ exhibited very low kinase activity, and its reactivation by the P1124L substitution was sensitive to every TKI (Figure 5F,G). Overall, these findings suggest a role for the DC2 in modulating TKI interaction with the ABL1 ATP binding pocket.

### 2.5. Deletion of the CC and DC2 Reduces BCR-ABL1 Transforming Potential in CD34+ Progenitors

**Figure 6:**
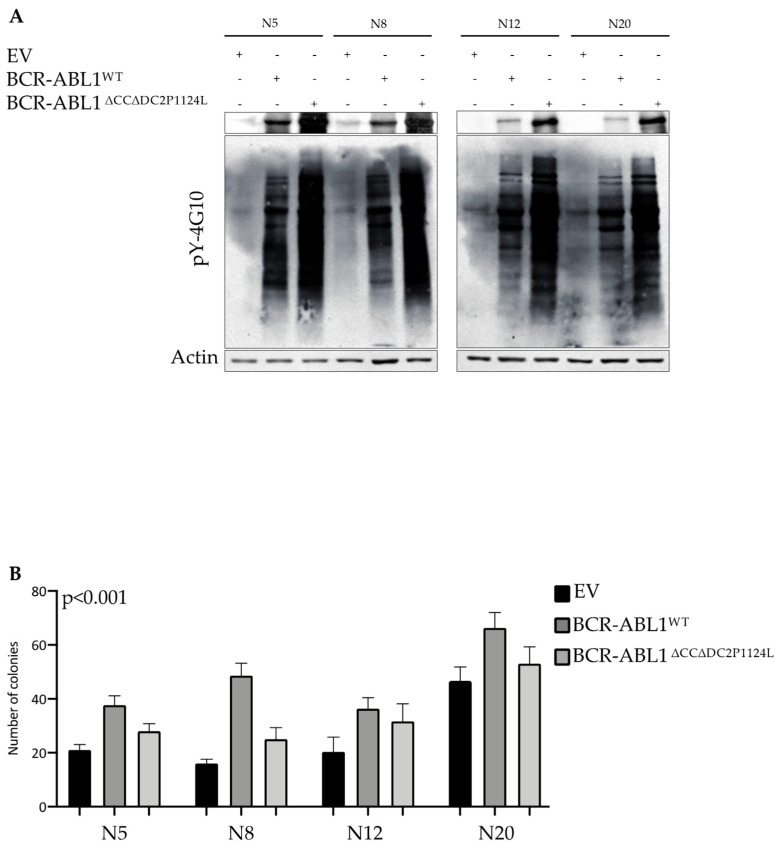
A monomeric kinase-proficient BCR-ABL1 isoform devoid of the C2 domain shows reduced CD34+ transforming potential compared to wild-type BCR-ABL1. (**A**) Protein lysates from human CD34+ progenitors lentivirally transduced with the indicated constructs were subjected to immunoblots to evaluate BCR-ABL1 expression (FLAG) and catalytic efficiency (pY-4G10). Actin was used as a loading control. (**B**) Cells reported in (**A**) were employed to perform colony-forming unit assays. Histograms indicate the number of colonies obtained after 15 days of culture. Bars represent the standard deviation of two independent experiments performed in duplicates. *p* values report the statistical significance by variance analysis (Anova) (* *p* < 0.05, ** *p* < 0.01, *** *p* < 0.001).

According to published data, the nuclear relocalization of the BCR-ABL1 oncoprotein induces cell death [9,11,12,13,33]. Although our previous experiments showed that the BCR-ABL1^∆CC∆DC2P1124L^ construct was capable of nuclear import, it failed to kill Ba/F3 cells (Figure 3 and Figure 4). To replicate this experiment in primary CD34+ progenitors, we transduced these cells with either BCR-ABL1^WT^ or BCR-ABL1^∆CC∆DC2P1124L^. When we analyzed the overall tyrosine phosphorylation levels, we found that the ∆CC∆DC2P1124L mutant induced higher intracellular tyrosine phosphorylation than BCR-ABL1^WT^ (Figure 6A). To evaluate if this kinase activation would result in colony formation, we implanted EV-transduced (as a control) BCR-ABL1^WT^ and BCR-ABL1^∆CC∆DC2P1124L^ CD34+ cells in methylcellulose and performed CFU assays. Compared to EV, BCR-ABL1^WT^ induced colony formation rates that were significantly higher than those detected in the absence of the CC and DC2 regions (Figure 6B). These observations suggest that—in human primary progenitors—BCR-ABL1-dependent leukemogenesis relies on both the oligomerization process and phosphoinositide interaction.

## 3. Discussion

Chronic Myeloid Leukemia is a myeloproliferative disorder characterized by the neoplastic transformation of the hematopoietic stem cell by the BCR-ABL1 oncoprotein [34]. We have previously shown the impact of the breakpoint region on the leukemogenic potential and the TKI responsiveness of BCR-ABL1 [35]. Here, we further expand these findings by demonstrating the involvement of two BCR domains on BCR-ABL1 intracellular localization, tyrosine kinase activity and TKIs’ sensitivity.

Nuclear-cytoplasmic shuttling is a complex molecular event that regulates the localization of multiple intracellular proteins. Indeed, modifications in the subcellular compartmentalization of several oncogenic drivers may alter cancer cell fate as well as response to therapy. Thus, nuclear–cytoplasmic shuttling currently represents an attractive therapeutic target investigated in different tumor types [36], as recently demonstrated by the Food and Drug Administration approval of the exportin 1 inhibitor selinexor for multiple myeloma [29]. Indeed, we and others have previously reported that targeting nuclear–cytoplasmic shuttling may provide additional therapeutic opportunities for patients diagnosed with Ph+ leukemias [10,37]. Nevertheless, the contribution of different BCR-ABL1 domains to the regulation of the oncoprotein’s intracellular localization and kinase activity remains partially unresolved.

Here, we demonstrate that BCR displays two NLS sequences which are functionally inhibited by the CC and DC2. The available data have shown that a functional NLS must be properly exposed to be recognized by importin alpha. Hence, both protein structure and protein–protein interactions might cause NLS masking [38]. Our analysis shows that a monopartite NLS is located in the BCR S/TK domain which contains two ABL SH2 binding sites [19] and several binding regions for 14-3-3 proteins [39,40]. The BCR bipartite NLS is located in the PH domain which reportedly interacts with membrane phosphoinositides [41]. Thus, we hypothesize that the activity of the two NLSs may be inhibited by the conformation of their flanking regions or by protein–protein interactions which may interfere with NLS-alpha importin contacts.

By generating a series of deletion constructs, we found that BCR^∆CC^ displayed a cytoplasmic localization, suggesting that oligomerization per se is not responsible for NLS masking. Indeed, combining the CC deletion with the removal of the C2 and Rho domains led to BCR nuclear accumulation, suggesting that these regions are responsible for NLS masking in the BCR protein (Figure 2). When we repeated these experiments on BCR-ABL1, we found that the DC2 deletion restored the catalytic activity of a monomeric kinase-deficient BCR-ABL1^∆CC^. Interestingly, despite its kinase activity, BCR-ABL1^∆CC∆DC2^ localized to the nucleus and this nuclear import was preserved in Ba/F3 cells even in the presence of the P1124L substitution, suggesting that both the CC and C2 domains are critical to determine BCR-ABL1 intracellular localization. However, although both BCR-ABL1^∆CC∆DC2^ and BCR-ABL1^∆CC∆DC2P1124L^ showed nuclear localization, only the P1124L mutant displayed significant cytotoxicity after IM+LMB when compared to LMB alone.

These findings suggest a possible role for the DC2 in mediating BCR-ABL1-dependent leukemogenesis. Interestingly, a BCR-ABL1 mutant devoid of the DC2 (BCR-ABL1^∆DC2^) was kinase proficient but exhibited low transforming activity, supporting a pivotal role for the C2 domain in leukemogenesis. When we deleted both the CC and DC2 (BCR-ABL1^∆CC∆DC2^), the resulting construct displayed moderate kinase activity but no transforming effect (Figure 4). These findings are in line with previous evidence indicating that the inactivation of the GEF domain does not affect BCR-ABL1 kinase activity but impairs its transforming potential [42,43]. However, when we increased BCR-ABL1^∆CC∆DC2^ catalytic activity by introducing the P1124L substitution (BCR-ABL1^∆CC∆DC2P1124L^), we observed IL3-independent growth that did not translate in a high number of colonies (Figure 4). These findings indicate that BCR-dependent oligomerization is necessary for BCR-ABL1-targeting of the correct downstream pathways required for hematopoietic cell transformation. As the ABL1 SH3-SH2 region is also required for the correct activation of the BCR-ABL1 tyrosine kinase [28,44,45], it is conceivable that the deletion of the DC2 may structurally affect ABL1 SH3-SH2, altering the oncoprotein’s transforming potential.

Finally, we investigated the role of the CC and DC2 on BCR-ABL1 TKI responsiveness and CD34+ transforming potential. In terms of TKI sensitivity, the removal of the coiled coil region had no influence on TKI efficacy. On the contrary, the deletion of the C2 domain (BCR-ABL1^∆DC2^) significantly reduced sensitivity to the available TKIs except for nilotinib, probably due to the drug’s prolonged intracellular accumulation and residence time after binding to the BCR-ABL1 kinase domain [46,47]. Unexpectedly, the addition of the P1124 mutation increased TKI responsiveness, suggesting that alterations in the SH3-SH2 region may affect the structural conformation of the ABL1 ATP binding pocket, thereby influencing TKI affinity. As for the transformation of human primary CD34+ progenitors, cells expressing BCR-ABL1^∆CC∆DC2P1124L^ showed high intracellular phosphorylation but failed to achieve the colony-forming ability of BCR-ABL1^WT^, implying that, regardless of its kinase activity, BCR-ABL1 requires both the CC and DC2 to achieve its full transforming potential on primary human progenitors.

While our study further defines the mechanisms contributing to BCR-ABL1 cytoplasmic retention, several issues will need additional clarification.

According to previous findings [10,33,48], the nuclear entrapment of BCR-ABL1 induces apoptosis. However, in our experiments, we failed to detect cell death in the untreated condition, registering an increase in apoptosis only after exposure to LMB or the combination of LMB+IM. This observation implicates that the integrity of the BCR protein may be required for cell killing when BCR-ABL1 is localized in the nucleus. Indeed, the CC domain is necessary for BCR-ABL1 transformation [28] and nuclear localization, as suggested by Peng and colleagues [49]. They demonstrated that the inhibition of the interaction between the BCR-ABL1 CC and HSP90AB1 relocalizes BCR-ABL1 to the nucleus, triggering apoptosis in CML cells. Hence, if on the one hand removing the CC domain restores nuclear import, on the other hand a monomeric BCR-ABL1 may be ineffective in inducing cell death. We should also consider a quantitative hypothesis, i.e., that a certain threshold of nuclear BCR-ABL1 is required to induce cell death. In this scenario, only cotreatment with the nuclear export inhibitor LMB achieves the BCR-ABL1 nuclear concentration requested to trigger apoptosis.

A second issue is the inability of the BCR-ABL1^∆CC∆DC2P1124L^ mutant to induce cell death when expressed in CD34+ cells. These results are in line with those previously published by Allan et al. [11], where BCR-ABL1 failed to kill CD34+ cells even in the presence of IM+LMB. However, given the limited number of the primary CD34+ progenitors available, we could not evaluate the intracellular localization of BCR-ABL1^∆CC∆DC2P1124L^ before and after LMB+IM exposure. Further experiments are in progress to investigate this aspect.

A final question concerns the potential therapeutic implications of our results. Indeed, our findings suggest that a docking study [50,51,52,53,54,55] on the CC and DC2 domains could identify small molecules potentially capable of simulating the conformational modifications induced by the deletion of these two regions. In turn, this may provide additional therapeutic strategies to manipulate both BCR-ABL1 intracellular localization and kinase activity.

## 4. Materials and Methods

### 4.1. Cell Lines and Drugs

HeLa and Ba/F3 cell lines were grown in RPMI 1640 supplemented with 2 mM glutamine, 100 units/mL penicillin, 50 µg/mL streptomycin (all from Sigma-Aldrich, Darmstadt, Germany) and 10% inactivated fetal bovine serum (FBS) (EuroClone, Milan, Italy). For Ba/F3, RPMI 1640 was also supplemented with 10% conditioned WEHI-3B cell medium to support interleukin 3 (IL3)-dependent growth. All cell lines were purchased from DSMZ (Braunschweig, Germany). Tyrosine kinase inhibitors, imatinib (IM) and nilotinib (NIL), were provided by Novartis; dasatinib (DAS) was supplied by Bristol Myers Squibb while ponatinib (PON) was purchased from Selleckchem (Houston, Texas, USA).

### 4.2. CD34+ Progenitor Isolation, Culture, and Colony-Forming Unit Assays

CD34-positive cells derived from healthy donors were purchased by American Type Culture Collection (ATCC), and CFU assays were performed as previously described [8,35,56].

### 4.3. Generation of Plasmids, Lentiviral Vectors and Mutagenesis Reactions

Plasmids encoding EGFP-NLSs, EGFP-PH, EGFP-STK and BCR wild-type or deletion mutants were generated as follows. For EGFP-NLSs, NLS sequences were inserted at the C-terminal of EGFP using the pEGFPC1 (Clontech, San Jose, CA, USA) plasmid as a backbone. For all EGFP-plasmids, carrying the wild-type and mutant (mEGFP, lysines and arginines substituted with a glutamine residue) NLS sequence, the primers sequence is indicated in Supplementary Table S1. Each EGFP-NLS was ligated in the pcDNA3.1 (Thermofisher, Waltham, MA, USA) plasmid in a unique EcoRI restriction site and then the 5′-3′ direction screened by Sanger sequencing.

EGFP plasmids carrying the PH and S/TK domains were amplified from pcDNA3.1-FLAG-BCR (a gift from Jean Y. J. Wang, University of California San Diego, La Jolla, CA, USA).

BCR-FLAG wild-type and deletion mutants were generated inserting the FLAG sequence in the reverse primers for all constructs. Each PCR product was inserted in pcDNA3.1 using the EcoRI restriction site and then the 5′-3′ direction screened by Sanger sequencing.

The pLEX-pCMV-IRES-PAC lentiviral vector (pLEX-EV, empty vector Dharmacon, Cambridge, UK) was employed to obtain FLAG-BCR-ABL1^WT^, as previously reported, and used as a backbone to generate FLAG-BCR-ABL1^∆CC^, FLAG-BCR-ABL1^∆DC2^ and FLAG-BCR-ABL1^∆CC∆DC2^ lentiviral vectors. Each BCR-ABL1 mutant was amplified using specific primers inserting the Kozak and FLAG sequences in the forward primer. First, FLAG-BCR-ABL1^∆DC2^ was obtained by separately cloning the BCR and ABL portions of the construct. The BCR sequence was amplified and cloned in the pLEX empty vector using SpeI-NotI restriction sites obtaining pLEX-BCR. The ABL sequence was amplified and then cloned in pLEX-BCR using the NotI-MluI restriction sites. FLAG-BCR-ABL1^∆CC^ and FLAG-BCR-ABL1^∆CC∆DC2^ deletion mutants were generated using FLAG-BCR-ABL1^WT^ and FLAG-BCR-ABL1^∆DC2^ as a backbone and ligated in pLEX empty vectors using the SpeI-MluI restriction sites.

To generate the BCR-ABL1 deletion mutants carrying the proline–leucine substitution in position 1124 (P1124L), each above-reported construct was subjected to a mutagenesis reaction employing the QuikChange II Site-Directed Mutagenesis Kit (Agilent, Santa Clara, CA, USA) generating FLAG-BCR-ABL1^∆CCP1124L^, FLAG-BCR-ABL1^∆DC2P1124L^ and FLAG-BCR-ABL1^∆CC∆DC2P1124L^. Finally, the FLAG-BCR-ABL1^∆CC∆DC2^ deletion mutant was subjected to a second mutagenesis reaction to modify the R and K residues of the ABL1 NLSs in Q, to generate the FLAG-BCR-ABL1^∆CC∆DC2P1124_ABL-NLSm^ construct. All primer sequences are reported in Tables S5–S7.

### 4.4. Cell Transfection

HeLa cells were transiently transfected with EGFP, BCR and BCR-ABL1 constructs, using the Transit-LT1 reagent according to the manufacturer’s instructions (MirusBio, Madison, WI, USA).

### 4.5. Lentivirus Production and Transduction

Lentiviral particles were produced by the transient transfection of TLAHEK293t cells as previously reported [35], and CD34+ cell lines were lentivirally infected, as described [35]. For Ba/F3 cells, lentiviral particles were used at a multiplicity of infection of 10 by spin-inoculation at 1200 g, at 4 °C for 90 min with this procedure repeated twice every 24 h. Cells were maintained in RPMI 1640 supplemented with 10% IL3 for 72 h, and then exposed to 3.5 µg/mL of puromycin to obtain the desired resistant clones. After three days, viable cells were resuspended in IL3-free RPMI 1640 and cultivated for an additional 72 h. At this time, cells were used for the indicated experiments. Lentiviral transduction of CD34+ cells was performed, as previously reported [35].

### 4.6. Immunofluorescence

For HeLa cells, immunofluorescence experiments were performed as reported for Ba/F3 cells, except for the poly-L-lysine stage. For Ba/F3 cells, coverslips were coated with a 0.01% poly-L-lysine (Sigma Aldrich, Darmstadt, Germany) for 10 min at room temperature. At this time, poly-L-lysine was removed, cells were resuspended in phosphate-buffered saline (PBS) and applied onto coverslips for 60 min at room temperature. PBS was removed and a paraformaldehyde fixing solution (3.7% formaldehyde, 0.2% Triton-X in PBS) was added for 30 min at 37 °C. To minimize non-specific adsorption, cells were overlaid with blocking solution (1% of bovine serum albumin in PBS) for 30 min at 37 °C. For cell staining, the blocking solution was removed, and coverslips were overlaid with primary antibody (anti-FLAG-M2-F3165, Sigma Aldrich) for 1 h, washed with PBS and subsequently exposed to the appropriate secondary antibody (Alexa Fluor-594, Thermofisher) for an additional hour. Cells were washed with PBS and colored with Hoechst 33258 for 5 min. Primary and secondary antibodies were diluted in blocking solution. Epifluorescence microscopy was performed with an Olympus microscope. Images were digitally acquired with an Orca CCD camera (Hamamatsu, Hamamatsu City, Japan) and processed with the Image-Pro Plus software v4.0 (Media Cybernetics, Silver Spring, MD, USA). Both HeLa and Ba/F3 cells were exposed to IM (10 µM) and leptomycin b (LMB) (10 nM) alone or in combination, as previously described [10]. The nuclear/cytoplasmic distribution for BCR-ABL1 wild-type and deletion constructs was evaluated using the Image J software v1.54 by measuring the Raw Integrated Density values expressed as percentage of pixels for each selected area.

### 4.7. Nuclear-Cytoplasmic Subcellular Fractionation and Immunoblot

To assess the subcellular localization of the BCR wild-type and deletion mutants, both nuclear and cytoplasmic fractions were isolated from transiently transfected HeLa cells using the Qproteome Nuclear Protein Kit following the manufacturer’s instructions (Qiagen, Hilden, Germany). For immunoblots, cells were resuspended in Laemmli buffer, sonicated and denatured. For both experiments, the protein lysates were separated by SDS-PAGE and transferred on nitrocellulose membranes, which were then hybridized using the following antibodies: anti-phosphotyrosine (clone 4G10; Millipore, Milan, Italy), anti-actin (AC-15), anti-tubulin (sc8035) anti-hystone 2B (sc10808), anti-Sp1 (sc59), anti-FLAG-M2-F3165, anti-AKT (9272), anti-p44-42 mitogen-activated protein kinase (ERK1/2, 9102) and anti-STAT5 (9363). All antibodies were purchased from Cell Signaling, except clones sc59 (anti-Sp1) and F3165 (anti-FLAG-M2), which were from Sigma. Phospho-specific antibodies anti-pAKT-Ser473 (9271), anti-p44-42 mitogen-activated protein kinase (ERK1/2, Thr202–Tyr204, 9101) and pSTAT5-Y694 (clone 9351) were all from Cell Signaling. Appropriate horseradish peroxidase-conjugated secondary antibodies were used to detect the indicated proteins using the LiteAblot enhanced chemiluminescence reagent (EuroClone).

### 4.8. Soft-Agar Colony-Forming Unit Assay

A 1.2% IL3-free RPMI solution of methylcellulose (M0512, Sigma-Aldrich), supplemented with 10% fetal bovine serum, was implanted at a density of 1, 3 and 5 × 10^3^ Ba/F3 cells ectopically expressing the indicated BCR-ABL1^WT^ or deletion mutants. After 10 days, colonies were counted under an optical microscope (IX71; Olympus, Milan, Italy).

### 4.9. IL3-Independent Growth

EV, BCR-ABL1^WT^ and deletion mutants expressing Ba/F3 cells were deprived of IL3 for 24 h. At this time, 10 × 10^4^/mL cells were cultivated in RPMI 1640 devoid of IL3 or supplemented with 1% or 0.1% IL3. All cell lines were counted in a hemocytometer every 24 h, mixing 10 µL of the cell culture suspension with 10 µL of a 0.4% Trypan Blue solution.

### 4.10. MTS Assay

For 24 h, 4 × 10^5^ lentivirally transduced Ba/F3 cells were deprived of IL3 and implanted in triplicates in IL3-free RPMI or in the same media that had been supplemented with IL3 for EV Ba/F3. All lines were exposed to the indicated drug concentrations and viable cells were counted every 24, 48 and 72 h employing the CellTiter 96^®^ AQueous One Solution Cell Proliferation Assay (Promega, Milan, Italy).

### 4.11. Bioinformatic Prediction of NLS Sequences

The presence of putative NLSs in the BCR protein sequence was investigated employing the PredictNLS (https://bio.tools/predictnls, accessed 8 March 2023) and P-Sort (https://www.psort.org/, accessed 8 March 2023) online tools, as previously described [57,58,59].

### 4.12. Statistical Analysis

The Prism Software v8.0 was used to perform variance analysis (ANOVA) and unpaired one-tailed *t*-tests with 95% confidence intervals.

## 5. Conclusions

CML Leukemic Stem Cells (LSCs) display BCR-ABL1-independent survival and are therefore unresponsive to TKIs, potentially leading to disease relapse if pharmacological treatment is discontinued. Previously published data show that the manipulation of BCR-ABL1 catalytic activity modulates the oncoproteins’ intracellular localization, resulting in the selective killing of leukemic cells. Here, we show that BCR displays two nuclear localization signals functionally inactivated by the coiled-coil and C2 domains, and that BCR-ABL1 constructs devoid of these domains display reduced transforming potential in Ba/F3 cells and in primary human CD34+ progenitors. The deletion of the CC and C2 domains also compromises TKI efficacy. In summary, by structurally manipulating several BCR domains, we have further defined the role of the BCR CC and DC2 in modulating BCR-ABL1 intracellular localization, transforming activity and TKI sensitivity. These data may contribute to devising additional therapeutic strategies that may interfere with BCR-ABL1-driven leukemogenesis.

## Data Availability

Data are contained within the article or Supplementary Material.

## Supplementary Materials

The following supporting information can be downloaded at: https://www.mdpi.com/article/10.3390/ijms26146591/s1.
