# Supplementary material for: The Coiled Coil and C2 Domains Modulate BCR Localization and BCR-ABL1 Compartmentalization, Transforming Activity and TKI Responsiveness
Source: Int J Mol Sci. 2025 Jul 9;26(14):6591. doi: 10.3390/ijms26146591 (PMC12295760; doi:10.3390/ijms26146591)
Supplement: Supplementary file 1 [file ijms-26-06591-s001.zip › Sup_Fig1.pdf]

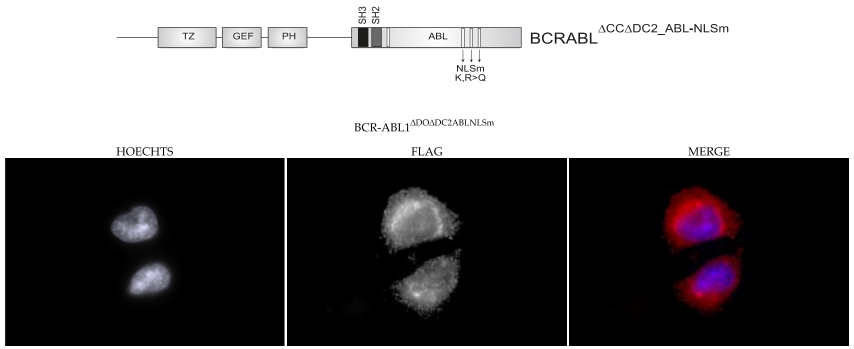

Supplemental Figure 1. The three ABL1 NLS do not modulate the nuclear import of BCR-ABL1$^{\Delta DO\Delta DC2}$. Immunofluorescence images obtained on HeLa cells showing nuclear (Hoechst) and BCR-ABL1$^{\Delta DO\Delta DC2ABLNLSm}$ (FLAG) staining.
