# Supplementary material for: The Coiled Coil and C2 Domains Modulate BCR Localization and BCR-ABL1 Compartmentalization, Transforming Activity and TKI Responsiveness
Source: Int J Mol Sci. 2025 Jul 9;26(14):6591. doi: 10.3390/ijms26146591 (PMC12295760; doi:10.3390/ijms26146591)
Supplement: Supplementary file 1 [file ijms-26-06591-s001.zip › TableS1_Revised.pdf]

**Supplemental Table 1.** Raw Integrity Density expressed as percentage of FLAG immunofluorescence staining obtained in both the Nuclear (N) and Cytoplasmic (C) compartments. SD: Standard Deviation.

| | ***RAW INTEGRATED DENSITY*** | | | | | | | | | | | | | |
|---|---|---|---|---|---|---|---|---|---|---|---|---|---|---|
| | BCR-ABL1 WT | | BCR-ABL1 ΔCC | | BCR-ABL1 $\Delta CC^{P1124L}$ | | BCR-ABL1 ΔDC2 | | BCR-ABL1 $\Delta DC2^{P1124L}$ | | BCR-ABL1 ΔCCΔDC2 | | BCR-ABL1 $\Delta CCD\Delta C2^{P1124L}$ | |
| | UT | IM+LMB | UT | IM+LMB | UT | IM+LMB | UT | IM+LMB | UT | IM+LMB | UT | IM+LMB | UT | IM+LMB |
| N (%) | 1.6 | 42.6 | 51.4 | 49.4 | 26 | 37.6 | 29.4 | 66.2 | 36.3 | 41.1 | 60.3 | 58.1 | 44.6 | 84.3 |
| S.D. | (±0.02) | (±0.12) | (±1.33) | (±0.95) | (±1.29) | (±2.68) | (±1.35) | (±2.32) | (±2.56) | (±7.62) | (±2.08) | (±2.81) | (±3.11) | (±7.76) |
| C (%) | 98.4 | 57.4 | 48.6 | 50.6 | 74 | 62.4 | 70.6 | 33.8 | 63.7 | 58.9 | 39.7 | 41.9 | 55.4 | 15.7 |
| S.D. | (±1.04) | (±0.50) | (±0.67) | (±0.60) | (±2.14) | (±2.41) | (±0.87) | (±1.16) | (±4.73) | (±6.14) | (±0.55) | (±0.55) | (±3.72) | (±0.67) |
