# Supplementary material for: The Coiled Coil and C2 Domains Modulate BCR Localization and BCR-ABL1 Compartmentalization, Transforming Activity and TKI Responsiveness
Source: Int J Mol Sci. 2025 Jul 9;26(14):6591. doi: 10.3390/ijms26146591 (PMC12295760; doi:10.3390/ijms26146591)
Supplement: Supplementary file 1 [file ijms-26-06591-s001.zip › TablesS5-6-7.pdf]

Supplemental Table 5: EGFP Primers	5′-3′ Sequence	Restriction Site
**Fw: EGFP**	*CGGAATTCATGGTGAGCAAGGGCGAGG*	EcoRI
**Rv: EGFP-NLS1 monopartite**	*CGGAATTCTTACCGGTGCCGCTTCTTGTACAGCTCGTCCATG*	EcoRI
**Rv: EGFP-NLS2 bipartite**	*CGGAATTCTTACAGCTTCTTCTTCAGCCTCTCCGTAGCCTTGCTGCCCTTGTTCGCCCTCTTCTTGTACAGCTCGTCCATGC*	EcoRI
**Rv: EGFP-NLS SV40**	*CGGAATTCTTAAACCTTTCTTTTCTTTTTAGGCTTGTACAGCTCGTCCATG*	EcoRI
**Rv: mEGFP-NLS1 monopartite**	*CGGAATTCTTACTGGTGCTGCTGCTTGTACAGCTCGTCCATG*	EcoRI
**Rv: mEGFP-NLS2 bipartite**	*CGGAATTCTTACAGCTGCTGCTGCAGCTGCTCCGTAGCCTGGCTGCCCTGGTTCGCCTGCTGCTTGTACAGCTCGTCCATGC*	EcoRI
**Fw: EGFP-PH**	*CCGCTCGAGATGACGGTGAAGGGAGAG*	XhoI
**Rv: EGFP-PH**	*CGGAATTCTCTGAAACACTTCTTCTGCTGC*	EcoRI
**Fw: EGFP-S/TK**	*CGGAATTCATGCAGCGATGGGGCTTCCGG*	EcoRI
**Rv: EGFP-S/TK**	*GGGGTACCCTCCAAGCCCTTTTCCAAGTCC*	KpnI

Supplemental Table 6: BCR-FLAG Primers	5′-3′ Sequence	Restriction Site
**Fw: BCR-FLAG, BCR-FLAG$^{\Delta Rho}$ and BCR-FLAG$^{\Delta DC2 \Delta Rho}$**	*CGGAATTCATGGTGGACCCGGTGGGCTT*	EcoRI
**Fw: BCR-FLAG$^{\Delta CC}$ and BCR-FLAG$^{\Delta CC \Delta DC2 \Delta Rho}$**	*CGGAATTCATGCAGCGATGGGGCTTCCGG*	EcoRI
**Rv: BCR-FLAG and BCR-FLAG$^{\Delta CC}$**	*CGGAATTCCTACTTATCGTCGTCATCCTTGTAATCGACTTCGGTGGAGAACAGGATG*	EcoRI
**Rv: BCR-FLAG$^{\Delta Rho}$**	*CGGAATTCCTACTTATCGTCGTCATCCTTGTAATCGACCCCTGTCTGTTTTCGGGA*	EcoRI
**Rv: BCR-FLAG$^{\Delta DC2 \Delta Rho}$ and BCR-FLAG$^{\Delta CC \Delta DC2 \Delta Rho}$**	*CGGAATTCCTACTTATCGTCGTCATCCTTGTAATCGAAACACTTCTTCTGCTGCTCC*	EcoRI

Supplemental Table 7: BCR::ABL1-FLAG primers	5′-3′ Sequence	Restriction Site
**FLAG-BCR:ABL1$^{\Delta DC2}$**		
**Fw: pLEX-BCR-FLAG**	*GACTAGTGCCACCATGGATTACAAGGATGACGACGATAAGATGGTGGACCCGGTGGGC*	SpeI
**Rv: pLEX-BCR-FLAG**	*ATAAGAATGCGGCCGCCTCTGAAACACTTCTTCTGCTG*	NotI
**Fw: ABL**	*ATAAGAATGCGGCCGCGAAGCCCTTCAGCGGCCAG*	NotI
**Rv; ABL**	*CGACGCGTCTACCTCTGCACTATGTCACT*	MluI
**Fw: FLAG-BCR:ABL1$^{\Delta CC}$ and FLAG-BCR:ABL1$^{\Delta CC \Delta DC2}$**	*GACTAGTGCCACCATGGATTACAAGGATGACGACGATAAGCAGCGATGGGGCTTCCGG*	SpeI
**Rv: FLAG-BCR:ABL1$^{\Delta CC}$ and FLAG-BCR:ABL1$^{\Delta CC \Delta DC2}$**	*CGACGCGTCTACCTCTGCACTATGTCACT*	MluI
