# Supplementary figures and images for: The Coiled Coil and C2 Domains Modulate BCR Localization and BCR-ABL1 Compartmentalization, Transforming Activity and TKI Responsiveness
Source: Int J Mol Sci. 2025 Jul 9;26(14):6591. doi: 10.3390/ijms26146591 (PMC12295760; doi:10.3390/ijms26146591)

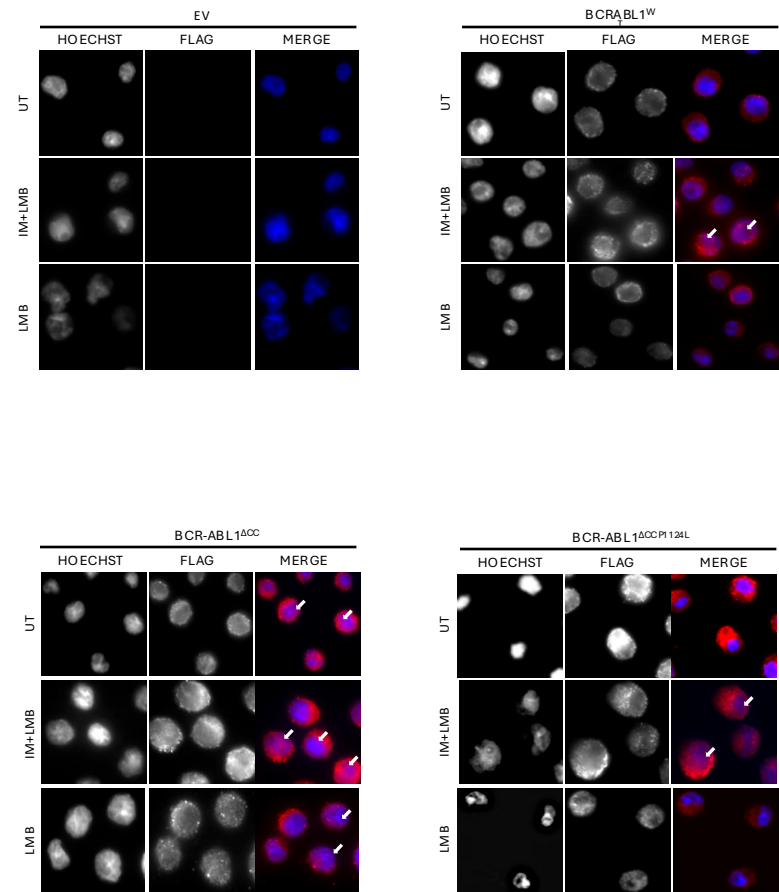

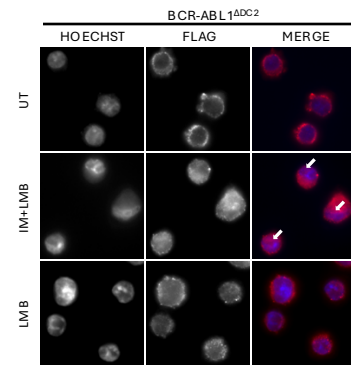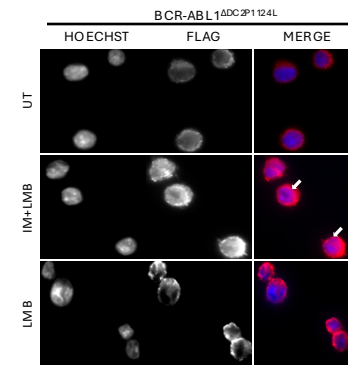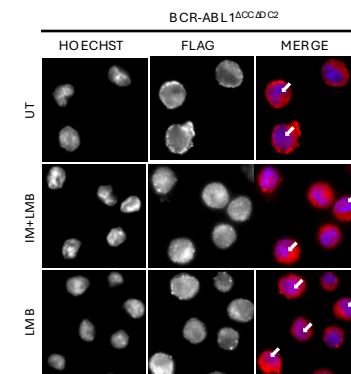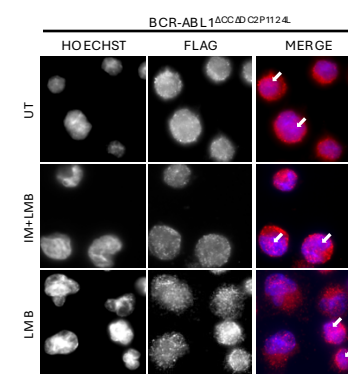

Supplement: Supplementary file 1 [file ijms-26-06591-s001.zip › Sup_Fig2.pdf]
